# The Proper Basis of Dental Charges

**Published:** 1870-05

**Authors:** C. A. Marvin


### DENTAL REGISTER.

VOL. XXIV.] MAY, 1870. [No. 5.

### Original Communications.

### THE PROPER BASIS OF DENTAL CHARGES.

BY C. A. MARVIN, D.D.S.

Read before the Brooklyn Dental Society.

MR. PRESIDENT AND GENTLEMEN:—The subject which I am to consider in this paper, is one of special interest to all who look upon Dentistry in its proper light, and desire to see it elevated to its appropriate place among the learned professions. What is the proper basis of Dental charges? In other words, what is to be taken into the account when making up a bill for Dental services?

The age in which we live is eminently a practical one. People act and think in a very matter-of-fact way. Tangible things—things that can be touched and handled—these are what are deemed valuable, while such as have no material form, though as truly real, are passed over as of little worth. People wish to see what they buy, and to know the exact value in dollars and cents of what they are expected to pay for. Everything presented for public acceptance must have a perceived value—a value that can be expressed in figures and reduced to items. The common demand is an equivalent— a *quid pro quo*, and this demand is not easily satisfied if the *quid* be an intangible matter. It must be present to the senses, something one can lay his hand on, can examine for himself, and learn if he has been cheated.

This is all very well in trade, when the transaction is merely the exchange of commodities for money, or of one commodity for another. One man has certain articles for sale, another wants them and is willing to give their value in money. Here is only an exchange and, therefore, the fair worth of the article is all that can of right be asked—a worth that can be estimated by any one. In fixing a price upon the article, the seller adds nothing for his superior judgment in buying or selecting his stock. The benefit of that judgment is already his, and lies in the superior character of his stock, or in its more advantageous purchase; making, in the first instance, his articles more desirable to the public, or in the second, leaving him a larger margin of profit when sold at current rates.

Now is this the proper rule to be observed by professional men when they ask for their compensation? Is the *quid* which they furnish to their patrons capable of being fully and minutely examined? Can it be itemized? Can it be put in such a tangible form that any one at a glance may fully appreciate its extent, understand its value, and pass a correct dollar-and-cent judgment upon it? Manifestly, no. Let us see what enters into the *quid* which a Dentist renders to his patient, and we shall then be better able to state what his *quo* should be.

You perceive, gentlemen, that I confine myself to the use of the common legal terms, and of course there is no difficulty in understanding my meaning. In the latin phrase, *quid pro quo*, as you all know, *quid* means services rendered, and *quo* the compensation demanded.

This *quid* then, what is it? It is complex. There enter into it, 1st, matured judgment; 2d, acquired skill; 3d, time; 4th, labor, and 5th, material.

All these are daily and hourly dispensed by every Dentist in full practice. Are they valuable? Let us see. Beginning, after the custom of the times, with the tangible, we will first consider the last element of those I have enumerated, viz: material.

Is material worth anything? But little time is necessary to answer this question. All will admit that material of whatever kind may be used, has some value, and this value is apparent. This being yielded, we have one item that should be charged for.

Next, labor. Is labor valuable? Every one will admit that it is. Especially will they who have recently built houses, or added any important improvements to those already built. So general is the belief that labor is valuable, that every snow shoveler, wood sawyer, coal heaver and carpet shaker at once names a price for his services in any of these capacities, almost startling. And this he does with such perfect composure, such an air of assurance that one feels it almost a violation of business courtesy to question the charge. From these humble occupations which I have named, up through all the gradations till we come to professional calling, do we find the same sentiment respecting labor, viz: that it is valuable. As we rise, however, we find the estimated value of labor increasing in regular ratio. For instance, the man who writes an essay in the public journal on agriculture, estimates the labor of his hands as more valuable than that of the ploughman who tills the soil. So the lapidary, who with delicate touch and exact eye, cuts the diamond, values his own labor higher than that of him who carves from wood the top of a gate post.

Here, then, appear two deductions, one of which we were seeking, viz: the general admission that labor is valuable. Having found this, we have a second element which should enter into our charge for Dental services.

The other deduction, resulting from the consideration of the last point is, that labor has different values in different avocations. The basis of this proposition is so intimately connected with one of the points I am to consider, viz: skill, that the examination of one will explain the other. Deferring it therefore, I take next in order the element of *time* as entering into the *quid* which a Dentist renders to his patient.

Is time valuable? Is life valuable? This earthly life of ours, with all its opportunities, its lessons, its responsibilities, is it of value? Then is time valuable, for time measures human life. If there be any value in human achievement, in human thought, in the influence of cultivated human minds, then is time valuable, for it has been largely expended in producing these. Time! it is a man's most valuable possession, to be cherished and husbanded and expended in the full appreciation of its worth. Think one moment. We can have it but once. An hour—a minute wasted, is gone forever. An hour gone! Where are we then? Our life is shortened by just one hour. We have one hour less to do our life's work in. We shall go to our graves with just one hour's work less to our credit, than if we had saved that wasted sixty minutes. Oh, is not time valuable? Bring it down to practical life. We have just so many hours in each day in which to work. We arrange our business so as to fill up all these hours. If tardiness on the part of a patient shortens the time of his appointment, how forcibly we realize the value of time when our operation not completed, the hour arrives for the next patient, and the patient with the hour. If we could only stretch time a little. If we could only increase the length of the hour, how it would help us. But that is impossible. On go the wheels, and steadily the day passes without cessation or possibility of recall.

As professional men, time forms a large part of our capital. We can not afford to lose it, nor give it away. When we expend it in the services of our patients we expect, we have a right to expect adequate compensation for it. We can spend the time only upon one person at once. And when the operations we are performing are completed, the time has gone and can never be again employed. Our hands we may use again. Our minds can be applied to the consideration of other cases. Our instruments and appliances are as useful for another operation as for the one just ended, but the time can not be recalled, can not be re-used. Once employed it is no more ours. It can yield but one revenue. It has been consumed by the patient who received our services, and that patient is responsible for it. We have expended it, it was valuable, and we should be paid for it. Here we have a third element entering into a proper bill for Dental services.

Next, acquired skill. What is it, and how attained? It is the direct and dexterous application of labor to the desired end. The end having been settled upon, the object to be attained clearly discerned, every act, every stroke, every direction, every expenditure of labor is aimed directly and without variation to that end. All the appliances in common use are made to do just what they were designed to do in the direction of the object to be accomplished. In the skilled hand, the instruments employed work regularly, progressively, and as if endowed with intelligence. The appliances to aid in the work, yield their service, take their allotted place and work for the one end. Under the skillful hand difficulties retire overcome, one by one; dangers which array themselves in threatening attitude, become entirely harmless; achievements that seem impossible of accomplishment at first view, are gradually and steadily approached and effected. There is no confusion, no waste of time, no useless outlay of labor. Every effort tells, system rules, and the intelligent observer is charmed by the orderly, confident and undeviating progress of the operation. This is skill, or skilled labor, as some call it. How is it attained? By long continued and toilsome practice. By close application and diligent study. By oft repeated trial. By failures and the strictest attention to the lessons which such failures teach. By consultation, by gathering up one by one the bits of useful information dropped in the conversation of older and expert operators. By reducing to practice every hint thus received. In these ways, and after years of patient and assiduous toil, the skill which the discriminating public to-day demands of our profession is attained. Is it valuable? Who will deny it? It is proved by the proposition before stated, that labor is differently valued in different avocations. Why is it so? Merely because of the different degrees of skill attending such labor. The involuntary verdict of those most inclined to underrate skill when considering it in the light of something to be paid for, is on the side of its value, for there is not one among them but would much quicker consign himself to the hands of the skillful principal than of the unskilled assistant.

Skill is valuable, and has been attained at great cost of time and labor, hence it is only reasonable that it should be paid for, thus we have a fourth element which should enter into a Dental bill.

Lastly, judgment. Is judgment worth anything? Why do parents go from one office to another to obtain opinions in the case of some important operation on their children's teeth? Why do they who sometimes employ him who works for the least money, wend their way at others to the most eminent in the profession? Why do parents who have confidence in the skill of their regular Dentist after receiving his opinion of a case presented, apply to another for his opinion of the suggestions of the first? Simply because they value judgment. They acknowledge its importance, and they desire its best expression as to the proper object to be aimed at in any given case.

Judgment takes precedence of skill. Judgment decides what is best to be done, and skill then takes hold and does it. Now, when we think how much research; how much and close observation; what laborious and protracted study; what delicate examinations; what careful comparisons precede and are necessary to a reliable judgment, we can form some adequate idea of its value. Things are valuable as they are useful to ourselves and others, and as they are difficult or expensive of procurement. On both these grounds judgment is valuable, and being so, when we expend it, it is right that we should be compensated.

To sum up then. In making his charge for Dental services the Dentist should take into the account judgment, skill, time, labor and material, and he should so fix his fee that none of these go unremunerated or forgotten.

